# A global dataset of microbial community in ticks from metagenome study

**DOI:** 10.1038/s41597-022-01679-7

**Published:** 2022-09-10

**Authors:** Mei-Chen Liu, Jing-Tao Zhang, Jin-Jin Chen, Ying Zhu, Bo-Kang Fu, Zhen-Yu Hu, Li-Qun Fang, Xiao-Ai Zhang, Wei Liu

**Affiliations:** 1 State Key Laboratory of Pathogen and Biosecurity, Beijing Institute of Microbiology and Epidemiology, Beijing, 100071 China; 2 School of Public Health, Anhui Medical University, Hefei, 230022 China; 3 School of Public Health, Wuhan University, 430000 Wuhan, China; 4 College of Life Sciences, Fujian Agriculture and Forestry University, Fuzhou, 350002 Fujian China

**Keywords:** Microbiome, Metagenomics

## Abstract

Ticks are important vectors of various zoonotic pathogens that can infect animals and humans, and most documented tick-borne pathogens have a strong bias towards microorganisms with strong disease phenotypes. The recent development of next-generation sequencing (NGS) has enabled the study of microbial communities, referred to as microbiome. Herein, we undertake a systematic review of published literature to build a comprehensive global dataset of microbiome determined by NGS in field-collected ticks. The dataset comprised 4418 records from 76 literature involving geo-referenced occurrences for 46 species of ticks and 219 microorganism families, revealing a total of 83 emerging viruses identified from 24 tick species belonging to 6 tick genera since 1980. The viral, bacterial and eukaryotic composition was compared regarding the tick species, their live stage and types of the specimens, or the geographic location. The data can assist the further investigation of ecological, biogeographical and epidemiological features of the tick-borne disease.

## Abstract (summary)

**Table:** 

Measurement(s)	microbial community
Technology Type(s)	Next Generation Sequencing
Factor Type(s)	tick
Sample Characteristic - Organism	tick
Sample Characteristic - Environment	microbial community
Sample Characteristic - Location	Whole world

## Background & Summary

Ticks are important vectors and reservoirs of a broad range of pathogens that are capable of causing diseases in humans, livestock, and wild animals. In the worldwide range, more than 800 tick species have been documented, including over 700 species in the family Ixodidae (hard ticks) and 193 species in the family Argasidae (soft ticks)^1,2^; At least 30 tick species are reported to feed on human beings and at least 103 known pathogens are transmitted by ticks^3–6^. Tick-borne pathogens co-evolve with their vectors and hosts and survive, multiply and circulate due to their adaptation to these different biological systems. Some are significant threats to human and animal health, for example, species of *Anaplasma*, *Babesia*, spotted fever group Rickettsiae, *Borrelia*, and viruses^3,6–8^. Ixodidae is the largest tick family having 3 active life cycle stages, including a single nymphal stage^9,10^. Argasidae also has 3 active life stages, but most species have multiple nymphal stages before developing into adults^4^.

Emerging and re-emerging tick-borne infectious diseases pose a continuing threat to human health. In the past three decades, application of molecular technologies had assisted in discovering new tick-borne pathogens and identifying the pathogenicity of the microorganisms previously detected in ticks. An increasing number of tick-borne pathogens have been reported, heartland virus^11^, tick-borne encephalitis virus^12^, *Borrelia burgdorferi* sensu lato^13^, *Rickettsia rickettsi*^14^, severe fever with thrombocytopenia syndrome virus^8^, are just a few examples of important pathogens that pose threats to human health. However, the diversity of tick-borne infectious diseases remained underestimated, since the investigations tended to be heavily biased toward research on microorganisms that infect humans or animals of economic and social importance^15,16^. The advent of advanced technologies such as high-throughput sequencing, meta-genomics, meta-transcriptomics, etc., had enabled a systematic understanding on a high variety of pathogenic or non-pathogenic, known or unknown, endogenous or exogenous microorganisms that are carried by ticks^9,15,16^. Several large-scale microbiome datasets derived from tick samples sourced from wide geographic regions are now publicly available in recent years. A number of novel tick-associated pathogens were discovered by NGS such as Bole Tick Virus 1, Changping Tick Virus 1, Dabieshan Tick Virus, Wuhan Tick Virus 2^17^. However, at present, a systematic account of microbiome data is lacking, thus far from adequate to attain a complete understanding of the diversity of tick-associated microbiome^15,16,18,19^.

Herein, we performed a systematic review of published literature to build a comprehensive global dataset on the diversity and distribution of microbiome by NGS performed in field-collected ticks. The data on the viral microbiome, bacterial microbiome and eukaryotic microbiome were assembled separately to identify all the viruses, bacteria and eukaryotes present in a tick sample, and to determine novel pathogens that can be carried by ticks.

## Methods

### Data collection

**Fig. 1:**
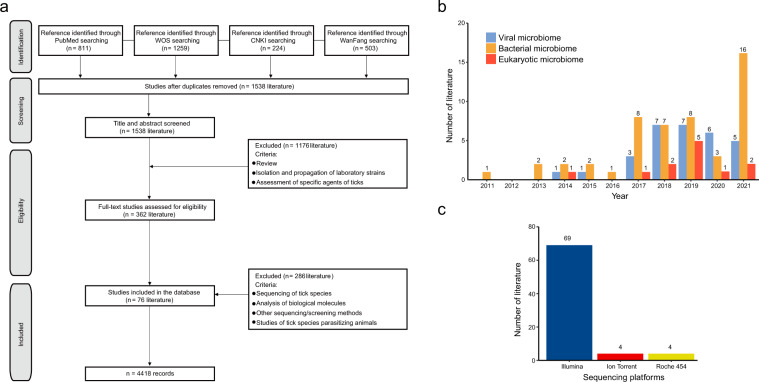
Schematic diagram of literature search. (**a**) Flow diagram on the literature search and screening process; (**b**) Annual number of literature that recorded field-collected ticks; (**c**) Number of literature grouped by the sequencing platform used. One literature evaluated the microbiome by using both Roche 454- and Illumina-based metagenomic approaches.

The study was performed according to the Preferred Reporting Items for Systematic reviews and Meta-Analyses (PRISMA) statement^20^. To attain an exhaustive review of the published literature on the microbiome diversity by NGS in field-collected ticks, a literature search was conducted on Chinese and English databases using a set of terms and Boolean operators, mainly through PubMed, Web of Science (WOS), China National Knowledge Infrastructure (CNKI) and the WanFang databases up to 1 April 2022, without language or publication-type restrictions. At the first step, general search terms were applied that included: “tick”, “*Amblyomma*”, “*Archaeocroton*”, “*Bothriocroton*”, “*Dermacentor*”, “*Haemaphysalis*”, “*Hyalomma*”, “*Ixodes*”, “*Nosomma*”, “*Rhipicephalus*”, “*Rhipicentor*”, “*Robertsicus*”, “*Antricola*”, “*Argas*”, “*Carios*”, “*Nothoaspis*”, “*Ornithodoros*”, “next-generation sequencing”, “high-throughput sequencing”, “deep sequencing”, “Roche 454”, “Illumina”, “Ion Torrent”, “SOLiD” in English literature databases search, and the keywords (“tick”, “virome”, “microbiome”, “metagenome”, “high throughput sequencing”, “deep sequencing”, “next generation sequencing”) were used in Chinese literature databases search. Data on all types of microorganisms, including viruses, bacteria, and eukaryotes were included. Emerging pathogens were defined as those first isolated or discovered after 1980. Ticks can feed on a wide range of vertebrates, therefore to highlight the presence of pathogens that were unique to ticks, we chose to include data that were performed on field-collected free-living ticks, while not include data from the detached ticks, since the latter might represent a complex microbiome of both tick and animal host derived. We excluded the following studies: (i) data obtained from experimentally fed ticks or detached ticks collected from animals; (ii) studies on the evaluation of the methods or the isolation and propagation of laboratory strains; (iii) review paper and (iv) studies that only tested the specific microorganism in ticks (Fig. 1a).

**Fig. 2:**
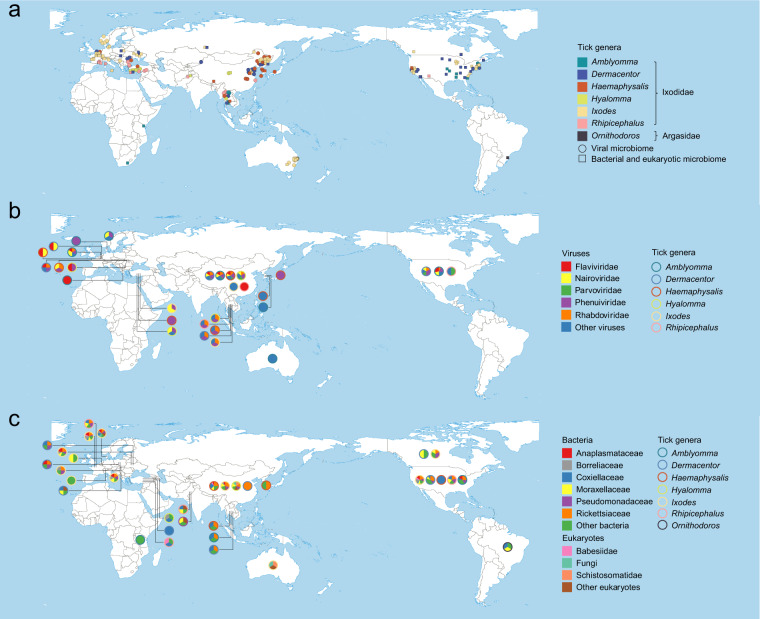
Geographical distribution of tick genus in relate to microbiome data at the province level. (**a**) Viruses, bacteria and eukaryotes; (**b**) Viruses; (**c**) Bacteria and eukaryotes.

A total of 2797 studies were retrieved for screening, comprised of 2070 from the English database and 727 from the Chinese database. The title and abstract of the retrieved studies were screened independently by three reviewers (MC L, JT Z, and Y Z) to identify studies potentially eligible for inclusion, which was narrowed down to 362 studies. For the third step, the full texts of the remaining studies were retrieved and independently assessed for eligibility by two reviewers (ZY H and BK F). Finally, a total of 7 Chinese and 69 English studies were eligible for data extraction (Fig. 1a). The earliest one was published in 2011, and the number of publications increased over the years, with a remarkable increase starting from the year 2017 (Fig. 1b). Of all selected studies, 69 (90.8%) used the Illumina sequencing platform, and 5.3% used the Ion Torrent sequencing platform (Fig. 1c). Data were from 46 species of ticks in 7 genera collected from 24 countries in 6 continents, and the geographical distribution of tick genera was shown in Fig. 2a. The viral metagenomic profiling, eukaryotic and bacterial microbiome profiling that corresponded to various tick genera were displayed across countries (Fig. 2b,c).

Full text of all the selected papers were reviewed, and data were extracted into a standardized dataset in Microsoft Excel 2019 that mainly includes: (i) identification of tested ticks at the family, genus, and species levels, (ii) methods for tick species identification, (iii) life cycle stages of the tested ticks, (iv) the geographic location of the ticks at country and province levels, (v) taxonomic annotations of microorganisms at family, genus, species levels, (vi) the platforms used for NGS. A re-check by two persons (MC L and JT Z) was performed to correct errors and remove duplicates. All conflicts of opinion and uncertainties were discussed and resolved by consensus with a third reviewer (JJ C). The main variable of interest was the viral/bacterial/eukaryotic component of the microbiome, determined for specific tick species at a specific site over time. All data were entered into the resultant by trained coauthors.

### Geo-positioning

The location information of the tick-collection site was extracted at the province level from the selected literatures. If no data on longitude or latitude were reported, or the location information was only given at a large scale such as a scenic area, mountainous region, ArcGIS 10.7 software was used to extract the geographical coordinates of the center points of the corresponding administrative areas from the digital map, which were obtained from GADM (Database of Global Administrative Areas) and Standard Map Service System. If the collection site could not be determined by any of these means, the authors were contacted for further information. We used R Studio Version 4.1.2 software and ArcGIS 10.7 software to statistically analyze and visualize the obtained geographic data.

## Data Records

The dataset of microbiome in field-collected ticks, based on NGS is available on figshare^21^. The columns contained in the dataset are shown as follows:

1. **ID**: Unique identifier code of the records.
2. **Tick families**: Identifies the family of tested ticks.
3. **Tick genera**: Identifies the genus of tested ticks.
4. **Tick species**: Identifies the species of tested ticks.
5. **Tick life cycle stages**: The developmental life stage of ticks (0 = Adult, 1 = Nymph, 2 = Larva, 3 = Not mentioned).
6. **Tick sex**: The sex of tested ticks (1 = Female, 2 = Male, 3 = Not mentioned).
7. **Identification methods**: Methods applied for identifying tick species (1 = Morphological identification, 2 = 16S rRNA sequencing, 3 = Other molecular diagnosis, 4 = Not mentioned).
8. **Microorganism types**: The types of microorganisms (1 = Viruses, 2 = Bacteria, 3 = Eukaryotes).
9. **Microorganisms**: Identification or initialism of microorganisms tested in the reference.
10. **Microbial families**: Identifies the family of determined microorganisms.
11. **Microbial genera**: Identifies the genus of determined microorganisms.
12. **Microbial species**: Identifies the species of determined microorganisms.
13. **Microbial taxonomy levels**: Taxonomy levels of determined microorganisms (1 = Family, 2 = Genus, 3 = Species, 4 = Other levels).
14. **Countries**: Collection site of tested ticks at the country level.
15. **Provinces**: Collection site of tested ticks at the province level.
16. **GPS_xx**: Longitude of reported province coordinates.
17. **GPS_yy**: Latitude of reported province coordinates.
18. **NGS platforms**: The sequencing platforms used in the study.
19. **References**: The full title of references used for data extraction.
20. **Publish time**: The year of publication.
21. **Collection time**: The year of tick collection.
22. **DOI**: The digital object unique identifier of references.

## Technical Validation

This dataset contains 4418 records that were extracted from 7 Chinese references and 69 English references. All recorded data were cross-checked by trained coauthors, and all uncertainties and discrepancies were discussed by consensus with a third reviewer. The first authors were also contacted to clarify the missing or ambiguous data.

The identification methods for tick species are critical in ensuring the credibility of the data, which is particularly relevant for juvenile stages when the morphological identification is difficult at the species level. The identification methods by morphology, molecular diagnosis for 18S rRNA sequencing, or combination of both methods were recoded. For the studies only used the morphological identification, the risk of confusion in tick species should be warned.

**Fig. 3:**
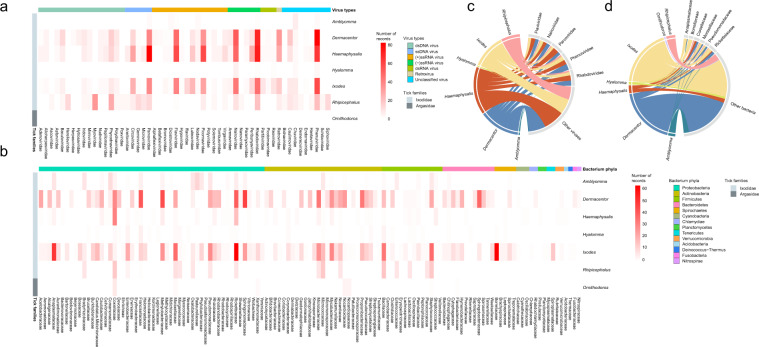
The viral and bacterial composition at phylum or family level, as well as the number of records that corresponded to the tick genus by heat chart (**a**,**b**) and by chord diagram (**c**,**d**).

In the process of verifying the geographic location of tick collection, an independent third-party was designated to re-check the information. The verification process refers to the same standard as that used in the data entry process. In order to unify the location information which provided no uniform standard to the province level, ArcGIS software was used to determine the coordinates of the central points of the provinces, which were marked on the Baidu Map to ensure that each coordinate point corresponds to an accurate administrative region. The geographic distribution of the ticks (*Amblyomma*, *Dermacentor*, *Haemaphysalis*, *Hyalomma*, *Ixodes*, *Rhipicephalus*, and *Ornithodoros*) that were tested for microbiome were separately displayed (Fig. 2a). The top five viral families (Flaviviridae, Nairoviridae, Parvoviridae, Phenuiviridae, and Rhabdoviridae) reported with the highest number of studies in relate to the tested ticks were mapped (Fig. 2b). The top five bacterial families (Anaplasmataceae, Coxiellaceae, Moraxellaceae, Pseudomonadaceae, Rickettsiaceae) reported with the highest number of studies in relate to the tested ticks, as well as Borreliaceae, the important tick-borne pathogens with a variety of vertebrates host and causes the most common tick-borne disease—Lyme borreliosis in the Northern Hemisphere^22,23^ were mapped (Fig. 2c). The two eukaryotic families (Babesiidae, Schistosomatidae) reported with the highest number of studies, as well as Fungi were mapped (Fig. 2c). The viral and bacterial composition at phylum or family level, as well as the number of records that corresponded to the tick genus were illustrated in Fig. 3. The (−)ssRNA fraction mainly consists of Rhabdoviridae (47.3%), Nairoviridae (35.8%), Peribunyaviridae (10.8%), Arenaviridae (3.6%), and Paramyxoviridae (1.8%) family, which together occupy 22.3% of the virome. (+)ssRNA viruses occupy 20.7% of the virome and mainly consist of members of the Flaviviridae (35.7%), Picornaviridae (31.1%), Luteoviridae (12.1%), Virgaviridae (5.6%), and Iflaviridae (3.2%) family.

Information about tick life cycle stages, tick genera, and sex were shown in Table 1. Of the 76 literature, the adult ticks, nymph ticks, and larva ticks were tested in 53, 23 and 9 of the literature, respectively. *Ixodes* was the most frequently tested tick genus (with totally 43 studies that underwent NGS), followed by *Dermacentor* (24). Female ticks were recorded in 53 studies, and male ticks in 47 studies.

**Table 1:** Number of literature determined microorganisms using NGS reported by life cycle stages, genera and sex of the ticks.

Characteristics	N	Tick life cycle stages			Tick genera							Sex	
		Adult	Nymph	Larva	*Amblyomma*	*Dermacentor*	*Haemaphysalis*	*Hyalomma*	*Ixodes*	*Rhipicephalus*	*Ornithodoros*	Female	Male
Total	76	53	23	9	11	24	22	7	43	9	1	53	47
Viruses													
Flaviviridae	12	6	0	0	1	5	5	0	9	1	0	6	5
Nairoviridae	14	8	3	2	0	0	5	2	9	2	0	8	8
Parvoviridae	5	3	0	0	1	2	2	0	1	1	0	2	3
Phenuiviridae	19	13	8	2	2	9	10	2	10	5	0	13	13
Rhabdoviridae	9	4	3	1	1	7	5	1	5	2	0	4	4
Other viruses	20	12	5	2	2	7	8	2	11	4	0	12	11
Bacteria													
Anaplasmataceae	17	10	4	0	2	1	1	0	13	0	0	10	5
Borreliaceae	15	9	3	0	0	0	0	1	14	0	0	9	7
Coxiellaceae	18	14	3	1	2	6	6	1	3	3	1	14	13
Moraxellaceae	19	14	5	3	0	3	2	2	11	2	1	14	11
Pseudomonadaceae	17	10	4	2	2	4	0	1	13	1	0	9	7
Rickettsiaceae	33	22	9	3	4	8	6	1	20	1	0	21	18
Other bacteria	45	34	14	4	8	11	6	3	24	3	1	34	30
Eukaryotes													
Babesiidae	6	3	1	0	0	0	1	0	4	1	0	2	2
Fungi	4	3	0	0	0	0	0	1	3	0	0	3	1
Other eukaryotes	6	5	1	0	0	1	0	2	3	1	0	5	4

Since 1980, a total of 83 emerging viruses were identified from 6 tick genera and 24 tick species by applying NGS. *Dermacentor nuttalli* and *Dermacentor silvarum* tick species harbored the highest variety of emerging viruses (26 species), followed by *Haemaphysalis concinna* (19) and *Dermacentor reticulatus* (13) (Table 2).

**Table 2:** Emerging viruses determined from tick species by applying NGS.

Tick species	Viruses
*Amblyomma americanum*	Lone star tick chuvirus 1, Lone star tick densovirus 1, Lone star tick dicistrovirus, Lone star tick nodavirus, Lone star tick totivirus
*Amblyomma testudinarium*	Mogiana tick virus
*Dermacentor marginatus*	American dog tick phlebovirus, Bole Tick Virus 3, Norway mononegavirus 1, Tacheng Tick Virus 3, Tick phlebovirus Anatolia 1, Wuhan tick virus 2
*Dermacentor niveus*	Stealth virus 1
*Dermacentor nuttalli*	American dog tick phlebovirus, Anguillid herpesvirus 1, Autographa californica multiple nucleopolyhedrovirus, Bovine viral diarrhea virus 1, Buenaventura virus, Chize virus, Columbid circovirus, Cotesia congregata bracovirus, Culex tritaeniorhynchus rhabdovirus, Drosophila melanogaster sigmavirus, Gata virus, Heliothis zea nudivirus, Jingmen tick virus, Kolente virus, Koolpinyah virus, Mogiana tick virus, Moumouvirus, Mucura virus, Murine leukemia virus, Pineapple bacilliform virus, Piper yellow mottle virus, Punta Toro virus, Sandfly fever Naples virus, South Bay virus, Wenzhou Tick Virus, Wuhan Louse Fly virus
*Dermacentor reticulatus*	American dog tick phlebovirus, American dog tick rhabdovirus-2, Bole tick virus 4, Changping Tick Virus 1, Changping Tick Virus 2, New Mapoon virus, Pacific coast tick phlebovirus, Pike fry sprivivirus, Tacheng Tick Virus 2, Tacheng Tick Virus 3, Taishun Tick Virus, Tick phlebovirus, Wesselsbron virus
*Dermacentor silvarum*	American dog tick phlebovirus, Anguillid herpesvirus 1, Autographa californica multiple nucleopolyhedrovirus, Bovine viral diarrhea virus 1, Buenaventura virus, Chize virus, Columbid circovirus, Cotesia congregata bracovirus, Culex tritaeniorhynchus rhabdovirus, Drosophila melanogaster sigmavirus, Gata virus, Heliothis zea nudivirus, Jingmen tick virus, Kolente virus, Koolpinyah virus, Mogiana tick virus, Moumouvirus, Mucura virus, Murine leukemia virus, Pineapple bacilliform virus, Piper yellow mottle virus, Punta Toro virus, Sandfly fever Naples virus, South Bay virus, Wenzhou Tick Virus, Wuhan Louse Fly virus
*Dermacentor variabilis*	American dog tick phlebovirus, American dog tick rhabdovirus-2, Severe fever with thrombocytopenia syndrome virus
*Haemaphysalis concinna*	American dog tick phlebovirus, Anguillid herpesvirus 1, Buenaventura virus, Chize virus, Cotesia congregata bracovirus, Culex tritaeniorhynchus rhabdovirus, Drosophila melanogaster sigmavirus, Gata virus, Heliothis zea nudivirus, Jingmen tick virus, Kolente virus, Koolpinyah virus, Moumouvirus, Mucura virus, Punta Toro virus, Sandfly fever Naples virus, South Bay virus, Wenzhou Tick Virus, Wuhan Louse Fly virus
*Haemaphysalis flava*	Hubei tick virus 3, Kabuto mountain virus
*Haemaphysalis formosensis*	Hubei tick virus 3
*Haemaphysalis hystricis*	American dog tick phlebovirus, Bole Tick Virus 3, Norway mononegavirus 1, Tacheng Tick Virus 3, Tick phlebovirus Anatolia 1, Wuhan tick virus 2
*Haemaphysalis longicornis*	American dog tick phlebovirus, Anguillid herpesvirus 1, Autographa californica multiple nucleopolyhedrovirus, Bovine viral diarrhea virus 1, Buenaventura virus, Chize virus, Columbid circovirus, Cotesia congregata bracovirus, Culex tritaeniorhynchus rhabdovirus, Dabieshan virus, Drosophila melanogaster sigmavirus, Gata virus, Heliothis zea nudivirus, Jingmen tick virus, Kolente virus, Koolpinyah virus, Mogiana tick virus, Moumouvirus, Mucura virus, Murine leukemia virus, Pineapple bacilliform virus, Piper yellow mottle virus, Punta Toro virus, Sandfly fever Naples virus, Severe fever with thrombocytopenia syndrome virus, South Bay virus, Wenzhou Tick Virus, Wuhan Louse Fly virus
*Haemaphysalis punctata*	American dog tick rhabdovirus-2, Bole Tick Virus 2, Bole tick virus 4, Brown dog tick phlebovirus 2, Nayun tick nairovirus, Taishun Tick Virus, Tick phlebovirus Anatolia 1, Wuhan House Fly Virus 1, Yongjia Tick Virus 2
*Hyalomma aegyptium*	Meram virus
*Hyalomma rufipes*	St Croix River virus
*Ixodes persulcatus*	American dog tick phlebovirus, Anguillid herpesvirus 1, Autographa californica multiple nucleopolyhedrovirus, Bovine viral diarrhea virus 1, Buenaventura virus, Chize virus, Columbid circovirus, Cotesia congregata bracovirus, Culex tritaeniorhynchus rhabdovirus, Drosophila melanogaster sigmavirus, Gata virus, Heliothis zea nudivirus, Jingmen tick virus, Kolente virus, Koolpinyah virus, Mogiana tick virus, Moumouvirus, Mucura virus, Murine leukemia virus, Pineapple bacilliform virus, Piper yellow mottle virus, Punta Toro virus, Sandfly fever Naples virus, South Bay virus, Wenzhou Tick Virus, Wuhan Louse Fly virus
*Ixodes ricinus*	Chimay rhabdovirus, Eyach virus, Gierle tick virus, Grotenhout virus, Jingmen tick virus, Norway mononegavirus 1, Norway luteo-like virus 1, Norway luteo-like virus 2, Norway luteo-like virus 3, Norway luteo-like virus 4, Norway nairovirus 1, Norway partiti-like virus 1, Norway phlebovirus 1, Zoersel tick virus
*Ixodes scapularis*	Avian-like circovirus, Blacklegged tick chuvirus-2, Blacklegged tick phlebovirus 1, Blacklegged tick phlebovirus 2, Blacklegged tick phlebovirus 3, Blacklegged tick picorna-like virus 1, Blacklegged tick rhabdovirus-1, Ixodes scapularis associated virus 3, Laurel Lake virus, New Kent County virus, Severe fever with thrombocytopenia syndrome virus, South Bay virus, Suffolk virus
*Rhipicephalus bursa*	Bole Tick Virus 3
*Rhipicephalus haemaphysaloides*	Dabieshan virus, Siniperca chuatsi rhabdovirus
*Rhipicephalus microplus*	American dog tick phlebovirus, Bole Tick Virus 3, Norway mononegavirus 1, Siniperca chuatsi rhabdovirus, Tacheng Tick Virus 3, Tick phlebovirus Anatolia 1, Wuhan tick virus 2
*Rhipicephalus sanguineus*	American dog tick phlebovirus, Bole Tick Virus 3, Brown dog tick phlebovirus 2, Jingmen tick virus, Siniperca chuatsi rhabdovirus, Tacheng Tick Virus 3, Tick phlebovirus Anatolia 1, Wuhan tick virus 2
*Rhipicephalus turanicus*	Bole Tick Virus 3

## Usage Notes

Investigating the potential tick-borne pathogens remains an important part of the source tracing and early warning of infectious diseases and emerging infections. To the best of our knowledge, this study represents the first attempt to comprehensively understand the microbial community, that was present in tick species acquired by using the NGS platform. NGS data from a total of 76 literature that recorded 46 species of ticks from 24 countries during 2011 to 2021 were compiled in the dataset. For each record, tick species were paired with relevant geo-positioning, timeline variables, microbiology composition, the number of records, and sequence platform. The dataset revealed the fundamental structure of the viral, bacterial and eukaryotic microbiome in tick species, which allowed for further comparative study. For example, the bacterial and viral composition of the NGS data could be compared regarding the tick species, their live stage and types of the specimens, or by their geographic location or collection season. The abundance of viruses or bacteria grouped at the family/genus/species level could be aligned, comparative analysis on the microbial community in ticks could be valuable. The data can also find out future application in the ecological, biogeographical and epidemiological study of the tick-borne disease, e.g., to investigate the occurrence of specific microorganisms in ticks; to the informed diagnosis of human patients with tick bites in different geographic regions.

## Supplementary information

Global dataset

## Data Availability

No custom code was made during the collection and validation of this dataset.
